# Visualizing CAR-T cell Immunotherapy Using 3 Tesla Fluorine-19 MRI

**DOI:** 10.1007/s11307-021-01672-3

**Published:** 2021-11-16

**Authors:** Veronica P. Dubois, Olivia C. Sehl, Paula J. Foster, John A. Ronald

**Affiliations:** 1 Robarts Research Institute, London, ON Canada; 2 The Department of Medical Biophysics, Western University, London, ON Canada; 3 Lawson Health Research Institute, London, ON Canada

**Keywords:** ^19^Fluorine magnetic resonance imaging, CAR-T cell therapy, Bioluminescence imaging, Cell tracking, Leukemia

## Abstract

**Purpose:**

Chimeric antigen receptor (CAR) T cell cancer immunotherapies have shown remarkable results in patients with hematological malignancies and represent the first approved genetically modified cellular therapies. However, not all blood cancer patients respond favorably, serious side effects have been reported, and the treatment of solid tumors has been a challenge. An imaging tool for visualizing the variety of CAR-T cell products in use and being explored could provide important patient-specific data on CAR-T cell location to inform on potential success or failure of treatment as well as off-target toxicities. Fluorine-19 (^19^F) magnetic resonance imaging (MRI) allows for the noninvasive detection of ^19^F perfluorocarbon (PFC) labeled cells. Our objective was to visualize PFC-labeled (PFC +) CAR-T cells in a mouse model of leukemia using clinical field strength (3 Tesla) ^19^F MRI and compare the cytotoxicity of PFC + versus unlabeled CAR-T cells.

**Procedures:**

NSG mice (*n* = 17) received subcutaneous injections of CD19 + human B cell leukemia cells (NALM6) expressing firefly luciferase in their left hind flank (1 × 10^6^). Twenty-one days later, each mouse received an intratumoral injection of 10 × 10^6^ PFC + CD19-targeted CAR-T cells (*n* = 6), unlabeled CD19-targeted CAR-T cells (*n* = 3), PFC + untransduced T cells (*n* = 5), or an equivalent volume of saline (*n* = 3). ^19^F MRI was performed on mice treated with PFC + CAR-T cells days 1, 3, and 7 post-treatment. Bioluminescence imaging (BLI) was performed on all mice days − 1, 5, 10, and 14 post-treatment to monitor tumor response.

**Results:**

PFC + CAR-T cells were successfully detected in tumors using ^19^F MRI on days 1, 3, and 7 post-injection. In vivo BLI data revealed that mice treated with PFC + or PFC − CAR-T cells had significantly lower tumor burden by day 14 compared to untreated mice and mice treated with PFC + untransduced T cells (*p* < 0.05). Importantly, mice treated with PFC + CAR-T cells showed equivalent cytotoxicity compared to mice receiving PFC − CAR-T cells.

**Conclusions:**

Our studies demonstrate that clinical field strength ^19^F MRI can be used to visualize PFC + CAR-T cells for up to 7 days post–intratumoral injection. Importantly, PFC labeling did not significantly affect in vivo CAR-T cell cytotoxicity. These imaging tools may have broad applications for tracking emerging CAR-T cell therapies in preclinical models and may eventually be useful for the detection of CAR-T cells in patients where localized injection of CAR-T cells is being pursued.

## Introduction

Cancer is a devastating disease accounting for nearly 10 million deaths globally in 2020 alone [1]. Despite enormous effort, cancer continues to be one of the leading causes of death in the world [2]. There continues to be an urgent need to develop new cancer therapies that allow for a greater number of cancer patients to survive for significantly greater lengths of time after their diagnosis. Chimeric antigen receptor (CAR) T cell therapy was first proposed in 1989 and is now the first genetically modified cellular therapy to be approved for the treatment of B cell leukemia and lymphoma [3]. CAR-T cells are produced using a patient’s own T cells that have been isolated and engineered to express a cancer antigen-specific CAR [4]. The CAR redirects the T cells to bind and kill the patient’s cancer cells after injection. Multiple CD19-targeted CAR-T cell therapies have been approved in Canada after showing remarkable results in patients with B cell malignancies, providing a transformative, potentially curative therapeutic option [5].

Despite the success that CAR-T cells show against B cell malignancies, major challenges remain. Up to 30% of blood cancer patients do not respond to these therapies, many patients can relapse, and patients can also suffer from life-threatening side effects such as cytokine release syndrome or neurotoxicity [6]. In addition, CAR-T cells continue to show disappointing results against solid tumors [7]. Many of the disparate outcomes among patients receiving this therapy are thought to be due to CAR-T cells not proliferating and persisting in the body, proliferating and activating excessively, or homing to normal organs such as the brain [8]. However, due to the inadequate information provided by serial blood tests currently used by clinicians, we have limited evidence about the behavior of CAR-T cells over time in individual patients. Therefore, methods to track the fate of adoptively transferred T cells would be extremely valuable for both preclinical and clinical studies to learn about the behavior of CAR-T cells after injection.

Cellular imaging is a potential complementary technology to blood tests involving noninvasive imaging of cells labeled with imaging technologies to achieve information on cell fate after adoptive transfer. Ex vivo labeling is a particularly invaluable approach for CAR-T cell therapies as they require ex vivo processing for production regardless of whether or not imaging is implemented. The breadth of cellular imaging technologies available spans from preclinical imaging modalities such as fluorescence and bioluminescence imaging (BLI) to clinical modalities such as magnetic resonance imaging (MRI) and positron emission tomography (PET) [9]. Importantly, successful PET imaging of intracranially infused cytotoxic T cells co-expressing a PET reporter gene was demonstrated in glioma patients [10, 11].

MRI is also being explored extensively as a clinical cell tracking tool. MRI provides images with fine spatial resolution and high soft tissue differentiation and uses non-ionizing radiation that can be beneficial for longitudinal studies, numerous MRI probes and reporter genes have been developed for ex vivo cell labeling, and MRI is broadly available within the healthcare system in most developed countries. Currently, most immune cell tracking studies utilizing MRI have been accomplished by labeling cells with superparamagnetic iron oxide nanoparticles (SPIONs) [12]. Clinical imaging of SPION-labeled dendritic cells in melanoma patients was achieved by De Vries et al*.* in 2005 [13]. SPIONs allow labeled cells to be detected with high sensitivity, even single cells in preclinical models, but detection of the cells can be difficult in locations such as the lungs as SPIONs cause hypointensities in images [14]. In contrast, fluorine-19 perfluorocarbons (PFCs) are a tracer agent that is easily taken up by cells and can be detected directly by fluorine-19 (^19^F) MRI [15]. In images acquired with ^19^F MRI, signal from PFC-labeled cells appears as a hot spot and is directly quantifiable. ^19^F MRI cell tracking has high specificity because there is no detectable endogenous ^19^F in the body [16]. In addition, ^19^F MRI has been successfully used in the clinic to detect PFC-labeled (PFC +) dendritic cells in patients [17]. Previous preclinical studies have shown the feasibility of labeling CAR-T cells with PFC and detecting them with high field strength ^19^F MRI [18–20]. However, data supporting the ability to image PFC + CAR-T cells using a clinical field strength scanner is lacking, which is important to demonstrate when assessing if translation of this technology into patients is feasible. Moreover, in vivo data comparing the cytotoxicity against tumors of CAR-T cells versus PFC + CAR-T cells is lacking.

In this study, we focused on using ^19^F PFC–based imaging to monitor CAR-T cells over time using clinical field strength 3 Tesla (T) MRI. In addition, we used bioluminescence imaging (BLI) to evaluate whether labeling CAR-T cells with PFC affects their in vivo cytotoxicity toward cancer cells. Our results indicate that this technique can reliably detect PFC + CAR-T cells post–intratumoral injection using clinical field strengths. We also show for the first time that PFC labeling does not significantly affect in vivo CAR-T cell cytotoxicity in a mouse model of leukemia, which is important for potential future use of this imaging technique in patients.

## Materials and Methods

### Cancer Cells and Engineering

A CD19-positive human B cell acute lymphoblastic leukemia cell line (NALM6 cells; Cedarlane) was utilized for this study. NALM6 cells were maintained in RPMI medium supplemented with 10% fetal bovine serum (Wisent) and 5 ml antibiotic–antimycotic (100 × ; Thermo Fisher). NALM6 cells were engineered to stably co-express the fluorescence reporter tdTomato (tdT) and a codon-optimized bioluminescence firefly luciferase reporter (Luc2) using a lentiviral vector previously constructed in our lab [21]. Cells were transduced with lentiviral vector using polybrene (1.6 µg/ml, Sigma Aldrich). Transduced cells were analyzed and sorted using fluorescence-activated cell sorting (FACSAria III flow cytometric cell sorter, BD Biosciences) and expanded prior to downstream use.

### Human T Cells and Engineering

Frozen peripheral blood mononuclear cells (PBMCs) from various donors were purchased from StemCell. PBMCs were cultured in ImmunoCult-XF T cell expansion medium (StemCell) supplemented with 100 U/ml interleukin-2 (Chiron) and 2 µL (55 µM) 2-mercaptoethanol (Sigma). T cell populations were obtained by thawing human PBMCs (StemCell) and activating 1 × 10^5^ cells per well with 2 µL of 4 × 10^7^ beads/ml human T-activator CD3/CD27 Dynabeads (Thermo Fisher) as outlined in the protocol from Hammill et al. [22]. Twenty-four hours later, T cells were engineered to co-express a CD19-targeted CAR and green fluorescent protein (GFP) using a CD19 CAR-GFP lentiviral transfer plasmid expressing a second-generation CD19 targeting CAR containing the 4-1BB co-stimulatory molecule generously gifted by Drs. Robert Holt and Brad Nelson (University of British Columbia) using an MOI of 5. Transduced and untransduced T cell populations were then expanded and evaluated with flow cytometry to evaluate CAR/GFP, CD3, CD4, and CD8 expression. To produce PFC-labeled (PFC +) CAR-T cells or PFC + untransduced T cells for ^19^F MRI, T cell populations were labeled overnight with 5 mg/ml Texas Red fluorescent dye conjugated PFC (CS-ATM DM Red, Celsense) and washed three times with phosphate-buffered saline (PBS) prior to downstream applications.

### Assessment of PFC Labeling

A cytospin of 100 × 10^3^ unlabeled and PFC^+^ CAR-T cells was prepared, and cells were then fixed in 4% paraformaldehyde for 5 min. Aqueous fluorescent mounting medium with DAPI (ab104139, Abcam) was used to identify CAR-T cell nuclei. Slides were imaged using a confocal Leica microscope (TCS SP8, Leica Microsystems) with a 63 × objective.

To evaluate the number of average ^19^F spins in cells, NMR was performed on samples containing 1 × 10^6^ PFC + CAR-T cells or PFC + untransduced T cells. To prepare the samples for NMR, the cells were lysed by adding 100 µL l radioimmunoprecipitation assay (RIPA) buffer (VWR, Mississauga, Canada), sonicated 3 times, and then underwent 3 freeze–thaw cycles. The lysate was then placed in an NMR tube with 0.1% trifluoroacetic (TFA) acid and heavy water (D_2_O) to a minimum volume of 600 µL l. ^19^F NMR measurements were performed using a Varian Inova 400 spectrometer (Varian Inc., Palo Alto, USA) as described by Makela et al*.* [23].

### *In Vitro* Imaging

To compare the cytotoxicity of PFC + and unlabeled CAR-T cells, 5 × 10^4^ NALM6-tdT-FLuc cells were seeded with PFC + or unlabeled CAR-T cells at increasing effector to target ratios (1:4, 1:2, 1:1). Twenty-four hours later, 1 µL of D-luciferin was added to each well (30 mg/ml, Syd Labs), and BLI was performed immediately on an IVIS Lumina XRMS scanner (IVIS Lumina XRMS In Vivo Imaging System, PerkinElmer). BLI signal was evaluated with region-of-interest (ROI) analysis using Living Image Software (PerkinElmer). Quantification was performed by drawing ROIs over each well to obtain the average radiance per well (photons/second/mm^2^/steradian).

To evaluate the minimum number of PFC + CAR-T cells that could be detected at 3 T, triplicates of CAR-T cell pellets containing decreasing numbers of labeled cells (2, 1, 0.5, 0.25, 0.1 (× 10^6^) cells) were imaged using ^19^F MRI. Samples were made by mixing labeled and unlabeled CAR-T cells to obtain a total of 2 × 10^6^ cells per Eppendorf tube; the Eppendorf tube was then spun down to form pellets and then covered with 1% agarose prior to MRI. The resulting samples were imaged at 3 T using a clinical GE 3 T MR750 system following the same imaging protocols used for in vivo imaging (see below). Analysis of all ^19^F MRI images is described further below.

### Animal Models

Animals were cared for in accordance with the standards of the Canadian Council on Animal Care, and under an approved protocol of the University of Western Ontario’s Council on Animal Care (2018–150). NOD.Cg-Prkdc^scid^Il2rg^tm1Wjl^/SzJ (NSG) mice (*n* = 17) received subcutaneous injections of 1 × 10^6^ NALM6-tdT-FLuc cells mixed with 50 µl of Matrigel in their left hind flank. Twenty-one days later, each mouse received an intratumoral injection of 10 x 10^6^ PFC + CAR-T cells (*n* = 6), unlabeled CAR-T cells (*n* = 3), PFC + untransduced T cells (*n* = 5), or an equivalent volume of saline (*n* = 3). The CAR-T cells were in 50 µL PBS and 50 µL Matrigel prior to injection. At the time of injection, the tumors were palpable.

### *In Vivo* BLI

BLI was performed on days -1, 5, 10, and 14 post-treatment in all mice. Mice were anesthetized with 2% isoflurane in oxygen during imaging sessions. Anesthetized mice received an intraperitoneal injection of 100 µL of D-luciferin (30 mg/ml), and images were collected using an IVIS Lumina XRMS scanner for up to 30 min. Day -1 images were used as a baseline for tumor burden to determine treatment response after CAR-T cell, T cell, or saline injections. BLI signal was evaluated with ROI analysis using Living Image Software (PerkinElmer). An ROI was drawn around the whole mouse and the total flux (photons/s) was measured to determine the peak signal in the 30-min imaging session. The peak signal for each mouse at each time point was recorded and used for statistical analysis.

### *In Vivo *^19^F MRI

Mice bearing leukemia tumors that received PFC + CAR-T cells or PFC + untransduced T cells were imaged with ^19^F MRI on days 1, 3, and 7 post-treatment. Mice were anesthetized with 2% isoflurane in oxygen during imaging sessions. ^1^H and ^19^F images were acquired on a clinical 3 T MRI (GE 3 T MR750 system, General Electric, ON, Canada) using a custom-built 4.3 × 4.3 cm^2^ dual tuned ^1^H/^19^F surface coil. In vivo ^1^H and ^19^F images were both acquired with a 3D balanced steady-state free precession (bSSFP) pulse sequence. Two reference tubes of PFC with known ^19^F concentration (3.33 × 10^16^ 19F/µl) were imaged alongside the mice for quantification purposes. ^1^H imaging parameters were the following: field of view (FOV) = 60 × 30 mm, matrix size = 150 × 75, slice thickness = 0.4 mm (0.4 × 0.4 × 0.4 mm^3^ resolution), flip angle (FA) = 20°, bandwidth (BW) = $\pm$ 31.25 kHz, repetition time (TR)/echo time (TE) = 12.8/6.4 ms, phase cycles (PC) = 12, total scan time = 9 min. ^19^F imaging parameters were as follows: FOV = 60 × 30 mm, matrix = 60 × 30, slice thickness = 1 mm (1 × 1 × 1 mm^3^ resolution), FA = 72°, BW = $\pm$ 10 kHz, TR/TE = 5.6/2.8 ms and 150 excitations (NEX), scan time = 27 min.

^19^F images were analyzed using Horos software. The standard deviation (Sdev) of background signal for each ^19^F image was measured by drawing a region of interest (ROI) in an area of background noise. A minimum threshold of 5 times the Sdev was used to mask lower amplitude signal and yield a reliable measurement of ^19^F signal in cell pellets, tumors, and reference tubes. This imaging criterion is based on an ^19^F signal with signal-to-noise (SNR) ratio > 5. Total ^19^F signal in cell pellets, tumors, and reference tubes was calculated as mean ^19^F signal × volume of ROI. ^19^F content in cell pellets and tumors was determined by comparing the ^19^F signal measured from these ROIs to the signal measured from the reference tubes (3.33 × 10^16 19^F/µl).

### Endpoint Histology

Two mice from the PFC + CAR-T cell treatment group and the PFC + untransduced T cell treatment group were euthanized via overdose of isoflurane on day 10 post-treatment. Their primary tumors were excised, fixed in 4% paraformaldehyde, and cryoprotected by passaging through a sucrose gradient of 10%, 20%, and 30%. Samples were then frozen in optimal cutting temperature compound using dry ice, and 10-µm sections were collected using a cryostat (Leica CM350 Cryostat). Tumor sections were stained with DAPI and imaged using fluorescence microscopy (EVOS FL Auto 2) to detect GFP expressing CAR-T cells.

### Statistics

Statistics were performed using the GraphPad Prism 8 Software. Unpaired *t*-tests were performed on the in vitro BLI cytotoxicity assay data to assess the difference between the cytotoxicity of PFC + and PFC − CAR-T cells. A simple linear regression was performed on the in vitro ^19^F MRI data to assess the correlation between ^19^F signal and cell number. A two-way ANOVA with multiple comparisons was performed on the in vivo ^19^F signal data to compare between the labeled treatment groups at each time point. A two-way ANOVA with Tukey’s multiple comparisons was performed on the in vivo BLI data to assess any differences in the treatment responses observed between treatment groups at each imaging time point. A nominal *p*-value less than 0.05 was considered significant.

## Results

### Production and Characterization of Treatment and Target Cells

**Fig. 1.**
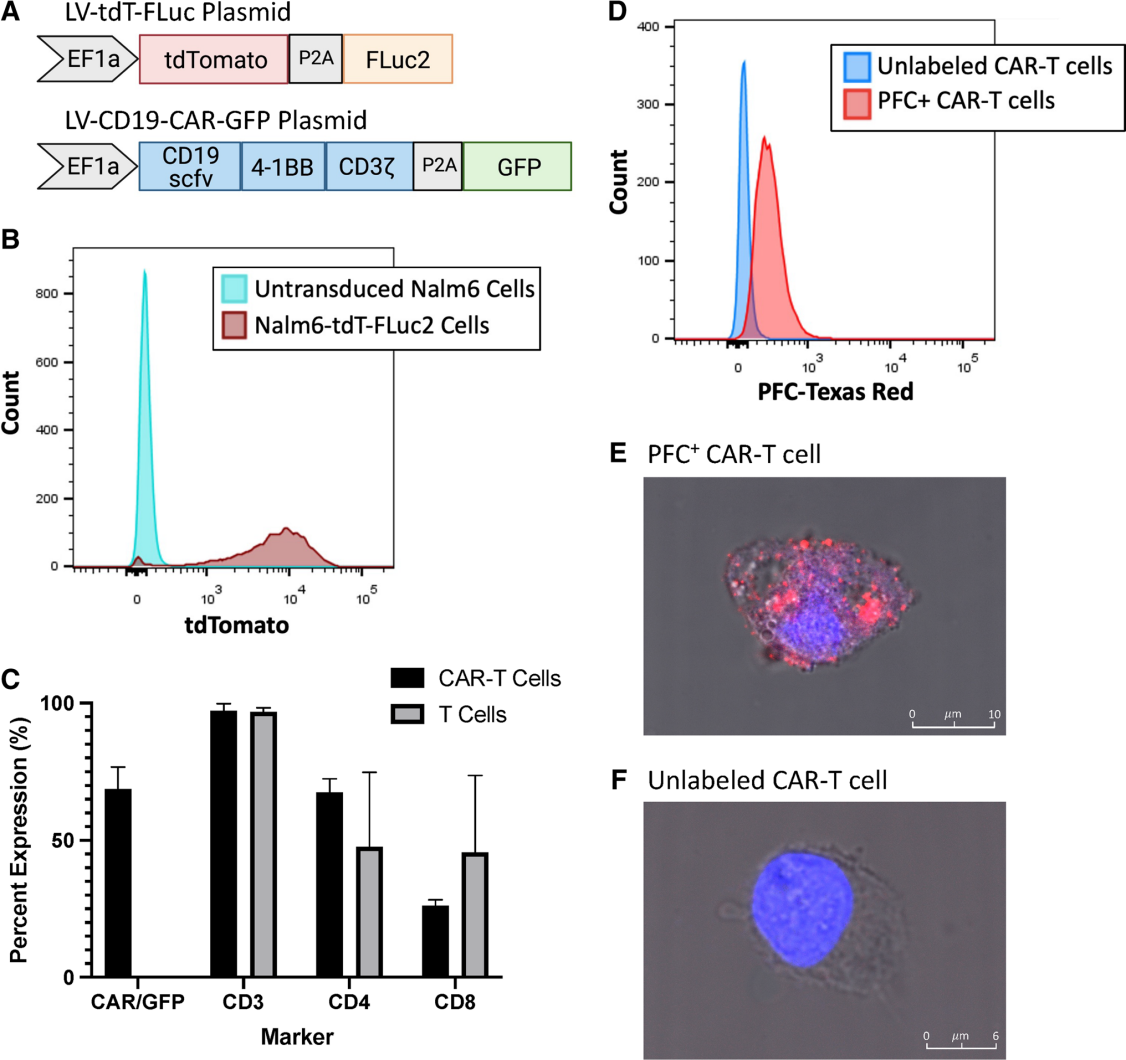
Production and characterization of CAR-T cells and their target cells. **a** Diagrams depicting a lentiviral (LV) tdT-FLuc transfer plasmid co-expressing the tdTomato fluorescent gene and firefly luciferase (FLuc2) bioluminescence reporter gene for the production of NALM6-tdT-FLuc cells and the LV-CD19-CAR-GFP plasmid co-expressing the CD19-targeted second-generation CAR and GFP for the production of CD19 CAR-T cells. **b** Merged histograms showing the NALM6 cell population before and after transduction with the tdT-FLuc lentivirus. **c** Bar graph showing the percent expression of CAR/GFP, CD3, CD4, and CD8 markers in CD19-CAR-GFP transduced T cell populations and untransduced T cell populations (*n* = 3). **d** Merged histograms showing a representative CAR-T cell population before and after labeling with red fluorescent perfluorocarbons. **d** Confocal microscopy shows intracellular localization of red fluorescence in PFC^+^ CAR-T cells and **e** unlabeled CAR-T cells do not exhibit red fluorescence. These images were merged with brightfield images to show the cell boundary, and blue fluorescence was used to identify CAR-T cell nuclei (DAPI stain).

Figure 1a shows a representation of the CD19-CAR-GFP plasmid used to make CD19 targeting CAR-T cells and the tdT-FLuc plasmid used to make firefly luciferase expressing NALM6 cells. Flow cytometry revealed that CAR-T cell populations were approximately 68.6% CAR/GFP positive after transduction. After transduction, 98.7% of NALM6 cells expressed tdT/Fluc (Fig. 1b). Further, the CAR-T cell populations were approximately 97% CD3 positive, 67.5% CD4 positive, and 26.1% CD8 positive prior to injection. Untransduced T cell populations showed similar characteristics with approximately 97% CD3-positive cells, 47.6% CD4-positive cells, and 45.5% CD8-positive cells (Fig. 1c). After labeling the T cell populations with 5 mg/ml Texas Red fluorophore–conjugated PFCs overnight, 88.8% of the cells were positive for uptake of PFC (Fig. 1d). Confocal microscopy was used to identify intracellular labeling of red fluorescent PFCs and the lack of red fluorescence in unlabeled CAR-T cells (Fig. 1e, f). As determined by ^19^F NMR, the labeled T cell populations contained 5.12 × 10^11 19^F/cell on average.

### *In Vitro* Assessment of PFC-Labeled CAR-T cells and Their Target Cells

**Fig. 2.**
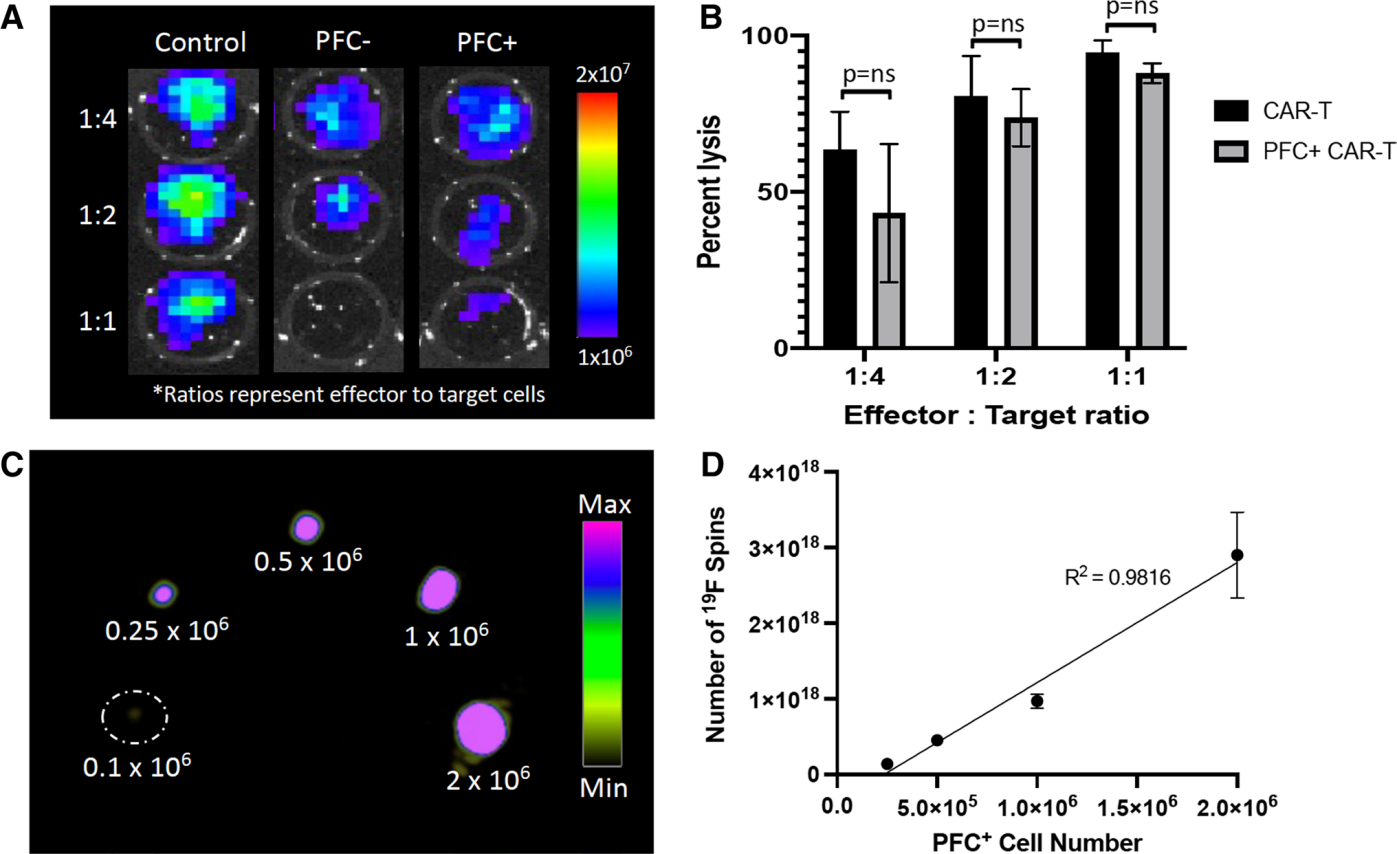
In vitro characterization of the PFC-labeled CAR-T cells. **a** BLI cytotoxicity assay showing the viability of NALM6-tdT-FLuc cells in the presence of saline (control), unlabeled CAR-T cells (PFC −), or PFC-labeled CAR-T cells (PFC +) at increasing effector to target ratios (*n* = 3). **b** Bar graph showing percent lysis of the NALM6-tdT-FLuc cells 24 h after treatment with PFC-labeled or unlabeled CAR-T cells. **c** ^19^F MRI of PFC-labeled CAR-T cell pellets (2 × 10^6^ total cells) containing decreasing numbers of labeled CAR-T cells (2, 1, 0.5, 0.25, 0.1 (× 10^6^) cells). **d** Quantification of ^19^F signal compared to cell number shows a strong positive correlation (*R*^2^ = 0.9816).

Figure 2 shows the in vitro characterization data for the imaging reporters and CAR-T cell cytotoxicity. BLI revealed that the NALM6-tdT-Fluc cell line had functional Fluc2 activity (Fig. 2a). Cytotoxicity assays showed that co-culture with unlabeled CAR-T cells caused an average of 63.6%, 80.5%, and 94.5% Nalm6-tdT-Fluc cell lysis at effector to target ratios of 1:4, 1:2, and 1:1, respectively (Fig. 2a). In comparison, PFC + CAR-T cells caused an average of 43.3%, 73.7%, and 90.0% Nalm6-tdT-Fluc cell lysis at effector to target ratios of 1:4, 1:2, and 1:1, respectively. There was no significant difference in cytotoxicity between unlabeled and PFC + CAR-T cells at any effector to target ratio (Fig. 2b). In vitro ^19^F MRI of PFC + CAR-T cell pellets showed that pellets containing only 2.5 × 10^5^ labeled CAR-T cells (2.31 $\pm$ 0.12 × 10^16 19^F spins/mm^3^, or 38.3 $\pm$ 2.0 mM ^19^F atoms) could be reliably detected at 3 T (*n* = 3). Quantification of the ^19^F spins revealed that there was a strong positive correlation between ^19^F signal and labeled cell number (*R*^2^ = 0.9816).

### ^19^F Cellular MRI Detection of PFC-Labeled T cells in Tumor-Bearing NSG Mice

**Fig. 3.**
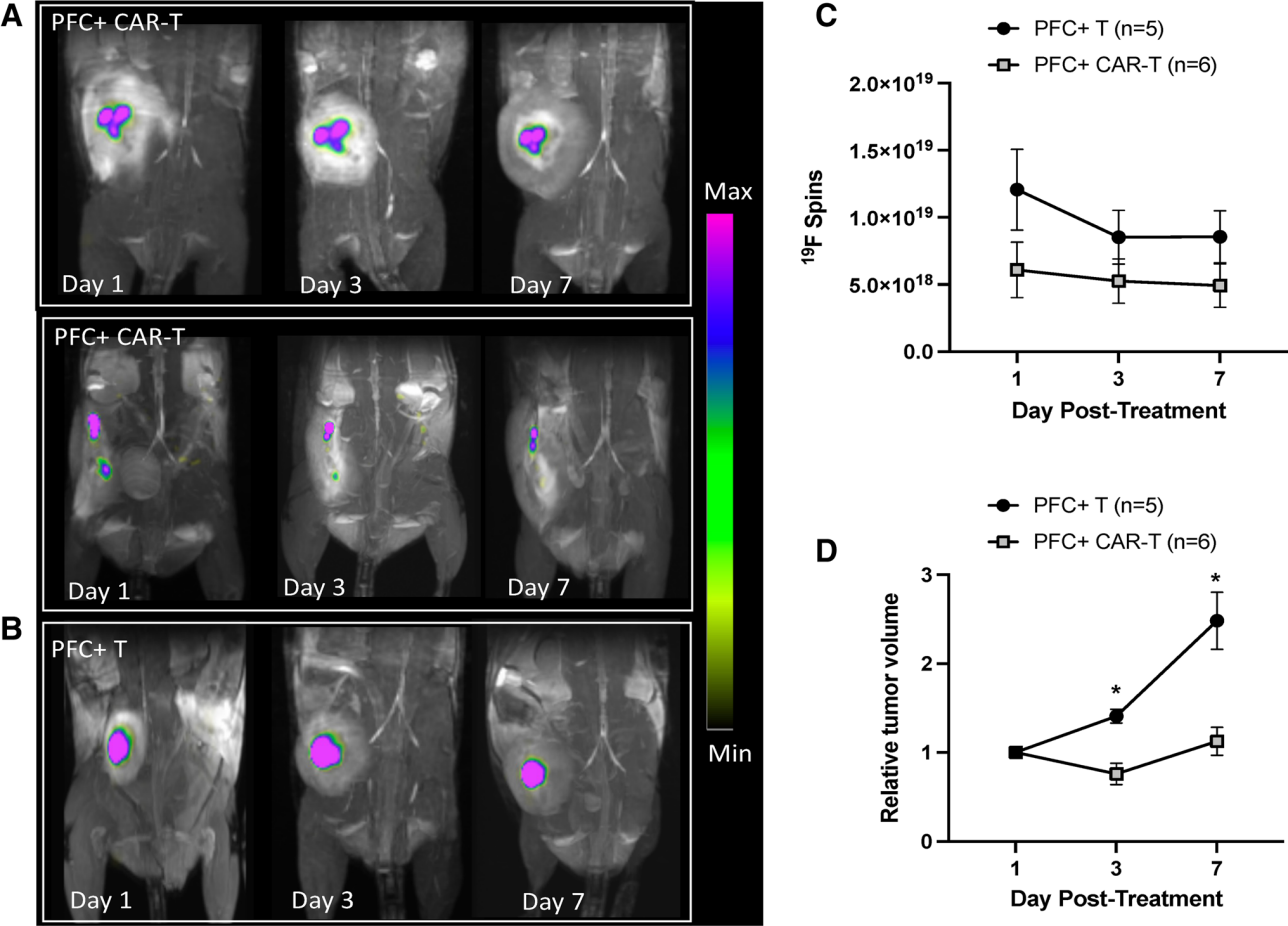
In vivo ^19^F MRI of leukemia tumor–bearing mice treated with 10 × 10^6^ PFC-labeled CAR-T cells (*n* = 6) or 10 × 10^6^ PFC-labeled untransduced T cells (*n* = 5). ^19^F images are overlaid onto ^1^H images for anatomical reference. ^19^F signal is detected in the tumors over time. Scale bars represent the range of ^19^F signals. **a** Representative images from two PFC-labeled CAR-T cell treated mice on days 1, 3, and 7 post-treatment. **b** Representative images of a PFC-labeled untransduced T cell–treated mouse on days 1, 3, and 7 post-treatment. **c** Quantification shows no significant differences of ^19^F signal over time between PFC + CAR-T cell and PFC + T cell groups. **d** Tumor volume significantly increases over time in mice that received untransduced T cells (* *p* < 0.05), but not in mice that received CAR-T cells.

In vivo detection of PFC + CAR-T cells using 3 T clinical MRI was assessed after intratumoral injections of 10 × 10^6^ cells into mice bearing subcutaneous Nalm6-tdT-Fluc tumors. All mice injected with NALM6-tdT-Fluc cells developed tumors in their left hind flanks by day 21 post-injection. Figure 3 shows representative ^19^F images of tumor-bearing mice after intratumoral injections of either PFC + CAR-T cells or PFC + T cells. ^19^F images are overlaid onto ^1^H images for anatomical reference. In all mice, ^19^F signal was present in the tumor on days 1, 3, and 7 post–PFC + cell injection (Fig. 3a and b). The ^19^F MRI data shows that the PFC + T cells and CAR-T cells were accurately injected intratumorally in all of the treated mice and that ^19^F signal is persistent at all time points. The total number of ^19^F spins for each tumor on days 1, 3, and 7 were quantified and are shown in Fig. 3c. The mean number of ^19^F spins was not significantly different between PFC + CAR-T cell–treated tumors and PFC + T cell–treated tumors at any time point.

### *In Vivo *BLI of Leukemia-Bearing Mice Treated with CAR-T cells

**Fig. 4.**
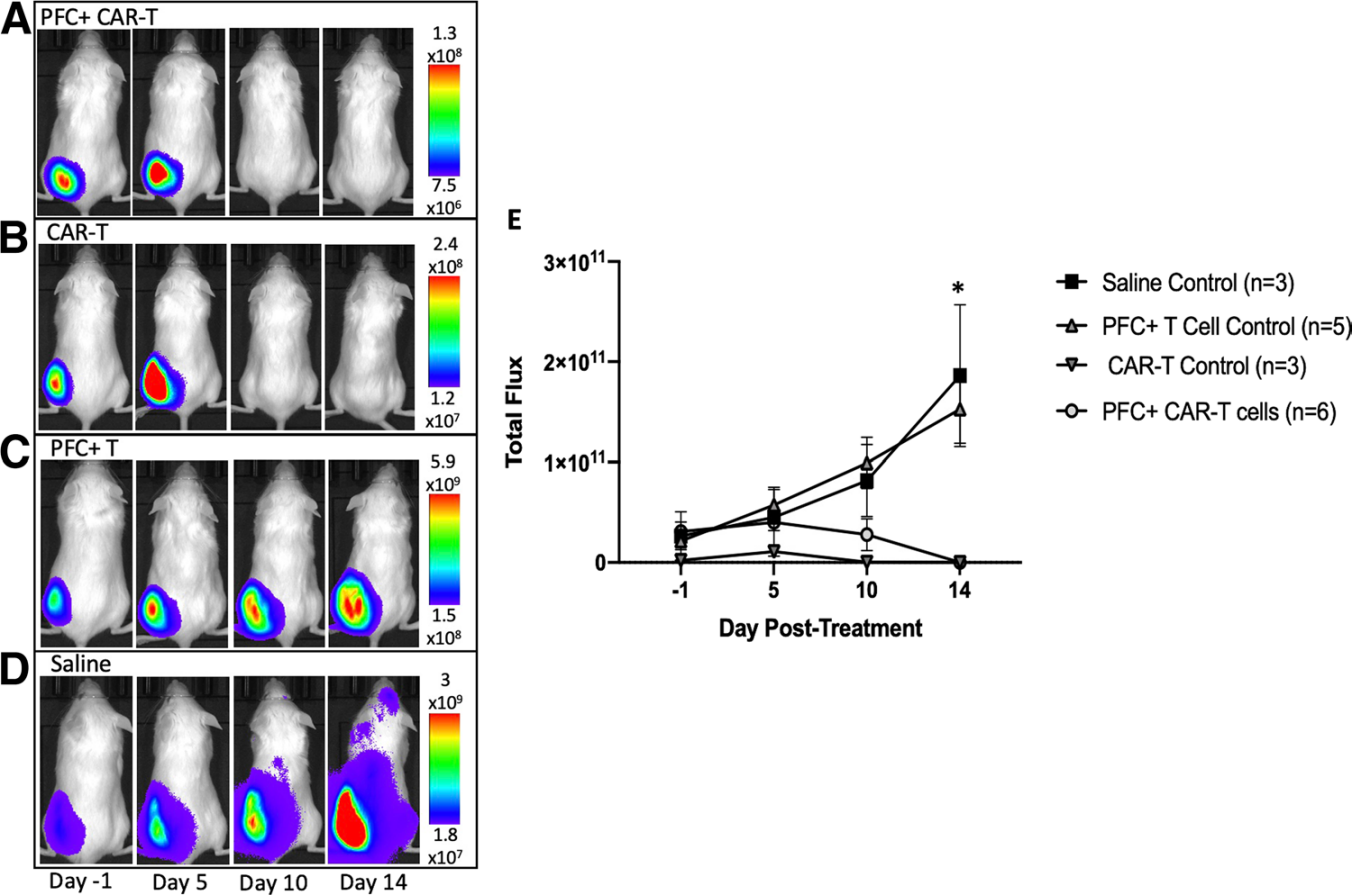
In vivo BLI of firefly luciferase expressing NALM6 tumor–bearing mice days − 1, 5, 10, and 14 post-treatment with intratumoral injections of 10 × 10^6^ PFC-labeled CAR-T cells (*n* = 6), unlabeled CAR-T cells (*n* = 3), PFC-labeled T cells (*n* = 5), or/and equivalent volume of saline (*n* = 3). **a** Representative images of a PFC-labeled CAR-T cell–treated mouse showing a decrease in tumor burden over time. **b** Representative images of an unlabeled CAR-T–treated mouse showing a decrease in tumor burden over time. **c** Representative images of a PFC-labeled T cell–treated mouse showing increases in tumor burden over time. **d** Representative images of a saline-treated mouse showing increases in tumor burden over time. **e** Quantitation of BLI signal over time showing significant differences between PFC-labeled CAR-T cell–treated mice compared to PFC-labeled T cell– and saline-treated mice on day 14 (*p* < 0.0001 and *p* < 0.0001). There is no significant difference between labeled and unlabeled CAR-T cell signal at any time point post-treatment.

To assess whether PFC labeling effected CAR-T cell therapy outcome, BLI images of NALM6-tdT-Fluc tumor–bearing mice were obtained up to 14 days after intratumoral injections of 10 × 10^6^ PFC + CAR-T cells (Fig. 4a), unlabeled CAR-T cells (Fig. 4b), PFC + T cells (Fig. 4c), or an equivalent volume of saline (Fig. 4d). PFC + and unlabeled CAR-T cell–treated mice showed decreased BLI signal after treatment. PFC + T cell– and saline-treated mice showed continuous increases in BLI signal after treatment. The total flux from each mouse at each imaging time point was quantified and is shown in Fig. 4e. Mice treated with PFC + CAR-T cells had significantly lower BLI signal by day 14 compared to mice treated with PFC + T cells or saline (*p* < 0.0001, *p* < 0.0001). There was no significant differences between the BLI signal in mice treated with saline or PFC + T cells at any time point. Importantly, there was no significant differences in BLI signal between mice treated with unlabeled CAR-T cells compared to mice treated with PFC + CAR-T cells at any time point.

### Endpoint Histology

**Fig. 5.**
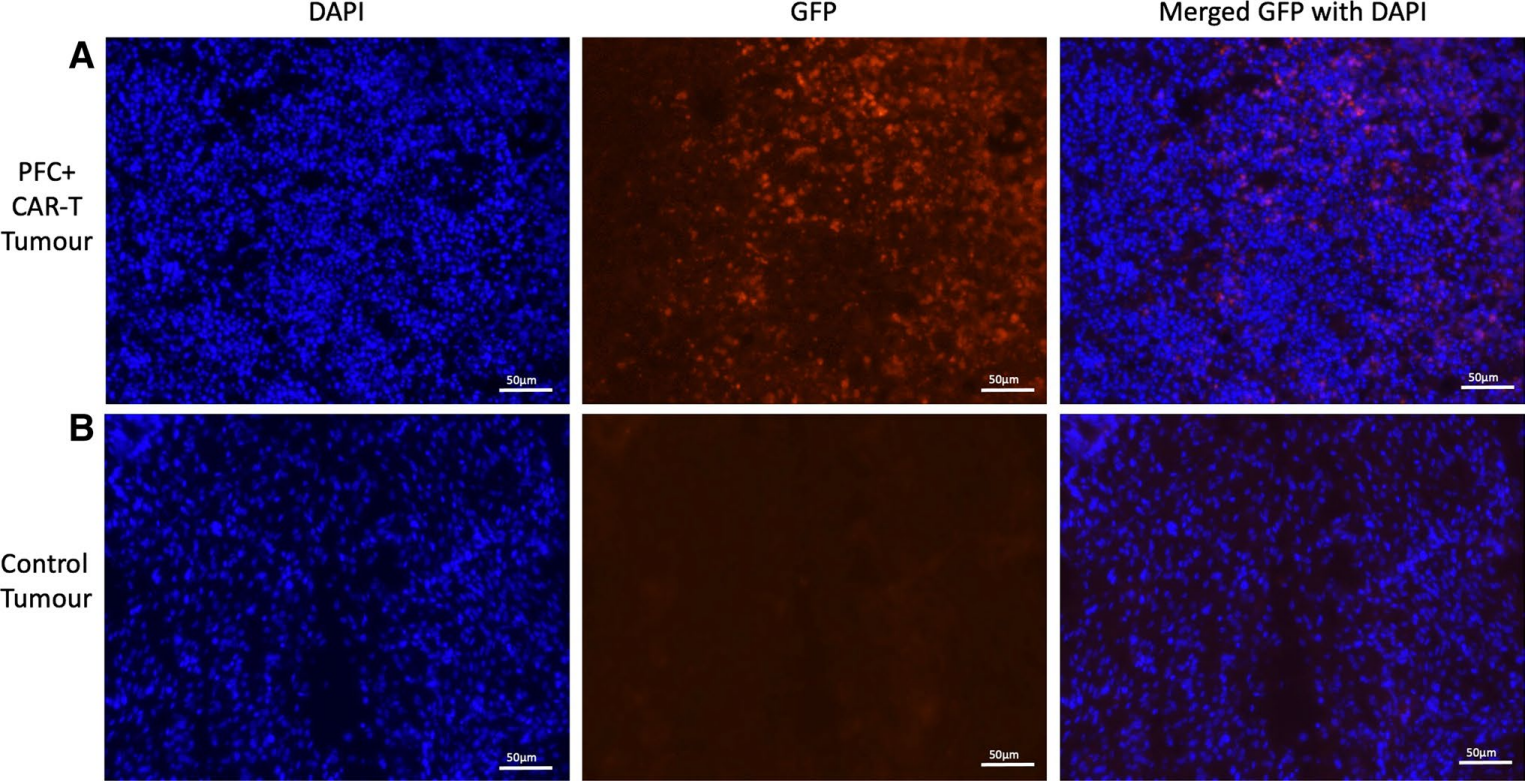
Histological analysis of GFP expression in a murine tumor treated with PFC + GFP expressing CAR-T cells or a murine tumor treated with PFC + untransduced T cells. **a** Images showing GFP-positive CAR-T cells in the tumor treated with PFC + CAR-T cells 10 days post–intratumoral injection. **b** Images showing no GFP-positive cells in tumors treated with PFC + untransduced T cells 10 days post–intratumoral injection. Images were taken at 20 × magnification.

Tumors from mice that received intratumoral injections of PFC + CAR-T cells or PFC + T cells were excised on day 10 post-treatment and analyzed to detect the presence or absence of CAR-T cells. Histological analysis confirmed that GFP-positive CAR-T cells were still present in PFC + CAR-T cell–treated tumors on day 10 post-injection (Fig. 5a). No GFP-positive cells was detected in tumors treated with PFC + T cells (Fig. 5b).

## Discussion

CAR-T cell therapies have shown tremendous promise in clinical trials against B cell malignancies. Despite these successes, there are still many limitations to overcome including overcoming their potential to cause serious side effects and increasing their efficacy in some patients with hematological malignancies and patients with solid tumors. Studies have shown that intratumoral injections of CAR-T cells may improve the treatment outcome in models of solid tumors [24, 25]. One of the main barriers in intratumoral treatments is ensuring that the injection is administered accurately to tumors that may be in different sizes and in different locations across patients. Acquiring adequate information on cell location and persistence after injection may improve intratumoral treatments by ensuring that each patient receives the therapy in the correct location. In this study, we demonstrate that ^19^F MRI at 3 T provides information on PFC-labeled CAR-T cell location and persistence after injection into mice bearing NALM6-tdT-FLuc tumors. Importantly, we saw no significant effect on CAR-T cell treatment due to PFC labeling.

Clinical translation of cell tracking requires a safe cell label that can be detected with imaging devices that are commonly found in hospitals. PET has been used in combination with clinically relevant radiotracers in many studies to track immune cells with high sensitivity [10, 26, 27]. Unfortunately, concerns about cost, half-life, and radioactive dose may limit longitudinal cell tracking studies using PET [28]. Although repeated PET imaging can be performed safely, the non-ionizing nature of MRI may prove to be advantageous for longitudinal cell tracking studies, particularly those studies where a significant amount of initial and follow-up scans may be required. For this reason, we chose to use ^19^F MRI as our imaging modality and ^19^F PFCs as our cell label because these are both clinically applicable and, in combination, allow for direct cell detection and quantification. CAR-T cells had been successfully labeled with PFCs for detection with ^19^F MRI in the past, but these studies used field strengths well above clinical field strengths to enhance 19F signal [18, 19]. In addition, these studies used CAR-T cells labeled with PFC nanoemulsions that are not, at this time, commercially available or manufactured in a manner acceptable for human use. We demonstrated that functioning CAR-T cells could be detected using 3 T clinical MRI using a surface coil and SNR optimized bSSFP sequence after labeling with commercially available PFC, which is also available in GMP form for clinical translation. Additionally, we were able to perform the imaging using a clinically feasible scan time of approximately 8 min for the ^1^H scan and 27 min for the ^19^F scan.

Our phenotyping results for PFC + CAR-T cells shown in Fig. 1 agree with previous studies showing that CD8 expressing cells make up approximately 1/3 of the population and CD4 expressing cells make up approximately 2/3 of the population [19, 20]. Our cell labeling allowed us to image down to 250,000 cells in vitro which agrees with previously published results suggesting that thousands of PFC + cells are needed per voxel to achieve detection [23]. Our intratumoral dose of 10 × 10^6^ cells was based on previous literature that used intratumoral doses of 8 × 10^6^ [29] and 1.5 × 107 CAR-T cells [30]. Our in vivo imaging of mice treated with PFC + T or CAR-T cells showed that ^19^F signal could be detected in every tumor on days 1, 3, and 7 post-treatment. Our findings on PFC + CAR-T cell detection after intratumoral injection are similar to recent results published by Chapelin et al*.* which looked at PFC + CAR-T cells up to day 10 post–intratumoral injection in a mouse model of glioma using an 11.7 T MRI scanner [18]. The ^19^F signal was consistent over time and suggested that the CAR-T cells were persisting in the tumor site. While we did show that CAR-T cells were present in tumors at day 10 post-injection, one limitation of our study was we were unable to demonstrate that the PFCs were still associated with these cells. Previous work by Chapelin et al*.* has shown that the PFC signal in glioma tumors was highly associated with CAR-T cells [20]. Moreover, while many CAR-T cells were still present at day 10, it is also possible that some of initial CAR-T cells have died, releasing the loaded PFCs, which might be cleared from the tumor or phagocytosed by tumor-associated macrophages, as seen with other imaging labels [31, 32]. We also did not see significant differences between the ^19^F signal detected in mice receiving PFC + CAR-T cells compared to PFC + T cells. This is consistent with their work and may be because T cells are surviving and remaining in the tumor site in both treatment groups.

We chose to complement our ^19^F MRI CAR-T cell detection with BLI to assess treatment response in our mice and determine if PFC labeling influenced in vivo CAR-T cell cytotoxicity. Our in vitro results showed no significant differences in cytotoxicity between labeled and unlabeled CAR-T cells, similar to previous work [18]. In addition, previous PFC + CAR-T cell tracking studies have shown that labeled CAR-T cells cause cytotoxicity against glioma in vivo. However, to our knowledge no studies have evaluated PFC + CAR-T cell in vivo cytotoxicity compared to unlabeled CAR-T cell cytotoxicity. BLI of luciferase-expressing tumors over time in mice treated with both PFC + CAR-T cells and unlabeled CAR-T cells demonstrated that PFC labeling does not significantly affect CAR-T cell in vivo cytotoxicity in this model.

There are still limitations to our cell detection method including ^19^F MRI being less sensitive compared to clinical imaging modalities such as PET and cell division preventing accurate measures of cell number over time [33]. These limitations are especially important when working with T cells because they are small and non-phagocytic which makes them more difficult to label [19, 34]. Nevertheless, in current clinical studies testing intratumorally injected CAR-T cells, patients receive up to 1 × 10^10^ CAR-T cells [35], which are well above the detection limit of ^19^F MRI [17, 23]. One advantage of ^19^F MRI is that it is quantitative and the number of cells in a given region can be estimated using in vitro NMR data to determine the amount of ^19^F per cell. This method can be used to quantify cell numbers early after injection. However, it is important to point out that this method is not as reliable for quantifying CAR-T cell numbers over time, as CAR-T cells have been shown to proliferate significantly after CAR interaction with their respective antigen [36]. During cell division, the PFC label should be divided between daughter cells. If these cells do not remain in the same voxels, this may decrease the ^19^F signal in an individual voxel below the detection limit, which would result in an underestimate of the number of CAR-T cells. Moreover, if the cells remain in the same voxel, this would still underestimate the number of CAR-T cells based on ^19^F spins. There is also the potential for background signal caused by macrophages taking up PFCs that are lost when labeled cells die after injection. However, studies indicate that when labeled cells die, the PFC is most likely broken down and released through the liver and then exhaled using the reticuloendothelial system [37]. Considering these limitations, it is therefore important to not overinterpret the ^19^F signal as the number of viable cells at extended periods after adoptive transfer, particularly in highly dividing cell populations. A complementary imaging tool such as reporter genes, which are passed to daughter cells, would allow for both highly sensitive short-term imaging with ^19^F PFCs and long-term cell viability imaging with a reporter gene [38, 39]. We are currently exploring the usefulness of this combination of cellular imaging technologies for tracking CAR-T cells in preclinical cancer models.

Currently, our system would be useful for detecting CAR-T cells after intratumoral injections into easily accessible tumors such as glioblastoma, metastatic colorectal cancer, and metastatic breast cancer [24, 25, 35]. It would be interesting to try imaging intravenously injected CAR-T cells in an animal model at clinical MRI field strengths in the future to determine if clinical field strength imaging of intravenously administered CAR-T cells would be feasible. Future work focusing on the development of larger radiofrequency coils for dual ^1^H and ^19^F MRI would also help advance this field. However, even if this is not feasible due to lack of sensitivity, it will still be valuable to continue to explore and develop ^19^F MRI of PFC-labeled CAR-T cells after intratumoral injections. In this case, localized coils with high sensitivity like the one used in our study would be valuable.

## Conclusions

We report that PFC + CAR-T cells can be detected over time with ^19^F MRI using a 3 T clinical field strength scanner. In addition, we show that PFC labeling does significantly impact the in vivo treatment response of CAR-T cells in this model, as shown by longitudinal BLI. ^19^F MRI is a useful tool for determining the location and persistence of CAR-T cells in tumors after localized injection. Future work will explore whether this tracking tool has utility for tracking systemically administered CAR-T cells in particular tumor types. This imaging tool may be useful for optimizing current CAR-T cell therapies and may have broad applications for evaluating emerging CAR-T cell formulations in vivo.
